# *S*-Ketamine oral thin film—Part 2: Population pharmacodynamics of *S*-ketamine, *S*-norketamine and *S*-hydroxynorketamine

**DOI:** 10.3389/fpain.2022.946487

**Published:** 2022-08-11

**Authors:** Pieter Simons, Erik Olofsen, Monique van Velzen, Maarten van Lemmen, Tom van Dasselaar, Patrick Mohr, Florian Hammes, Rutger van der Schrier, Marieke Niesters, Albert Dahan

**Affiliations:** ^1^Department of Anesthesiology, Leiden University Medical Center, Leiden, Netherlands; ^2^LTS Lohmann Therapie-Systeme AG, Andernach, Germany

**Keywords:** ketamine, *S*-ketamine, pharmacodynamics, antinociception, modeling, oral transmucosal film

## Abstract

Ketamine is a versatile drug used for many indications and is administered *via* various routes. Here, we report on the pharmacodynamics of sublingual and buccal fast-dissolving oral-thin-films that contain 50 mg of *S-*ketamine in a population of healthy male and female volunteers. Twenty volunteers received one or two 50 mg *S-*ketamine oral thin films in a crossover design, placed for 10 min sublingually (*n* = 15) or buccally (*n* = 5). The following measurements were made for 6 h following the film placement: antinociception using three distinct pain assay; electrical, pressure, and heat pain, and drug high on an 11-point visual analog scale. Blood samples were obtained for the measurement of plasma *S-*ketamine, *S*-norketamine, and *S*-hydroxynorketamine concentrations. A population pharmacodynamic analysis was performed in NONMEM to construct a pharmacodynamic model of *S*-ketamine and its metabolites. *P*-values < 0.01 were considered significant. The sublingual and buccal 50 and 100 mg *S-*ketamine oral thin films were antinociceptive and produced drug high with effects lasting 2–6 h, although a clear dose-response relationship for antinociception could not be established. The effects were solely related to the parent compound with no contribution from *S*-norketamine or *S*-hydroxynorketamine. *S*-ketamine potency was lower for antinociception (C_50_ ranging from 1.2 to 1.7 nmol/mL) than for drug high (C_50_ 0.3 nmol/ml). The onset/offset of effect as defined by the blood-effect-site equilibration half-life did not differ among endpoints and ranged from 0 to 5 min. In conclusion, the 50-mg *S-*ketamine oral thin film was safe and produced long-term antinociception in all three nociceptive assays with side effects inherent to the use of ketamine. The study was registered at the trial register of the Dutch Cochrane Center (www.trialregister.nl) under identifier NL9267 and the European Union Drug Regulating Authorities Clinical Trials (EudraCT) database under number 2020-005185-33.

## Introduction

The *N*-methyl-D-aspartate receptor antagonist ketamine experiences a clinical renaissance due to the introduction of various new indications (1). While initially developed as an anesthetic and a substitute for phencyclidine, it later gained popularity as an analgesic and currently is available as a rapid-acting antidepressant (1). Ketamine has multiple administration routes that may be divided into those that require a sometimes painful injection or venipuncture (intravenous and subcutaneous delivery) and those that circumvent the disadvantages of delivery by injection and allow an easy and painless treatment. The latter route includes delivery by oral, intranasal, transcutaneous, or rectal routes. Here, we study the pharmacodynamics [and in part 1 of this study, the pharmacokinetics, see the accompanying paper (2)] of sublingual and buccal fast-dissolving oral-thin-films (OTFs) that contain 50 mg of *S-*ketamine, one of the isomers of ketamine. The OTF is a rectangular 4.5 cm^2^ thin film that is loaded with an active substance that immediately dissolves in the mouth and is rapidly absorbed through the mucosa. Ketamine is a complex drug for various reasons; it is a racemic mixture of *S-* and *R-*isomers and is metabolized into active compounds such as norketamine and hydroxynorketamine (3, 4). All these differ in pharmacokinetics and dynamics, and consequently may influence the ultimate effect of the drug and consequently the *S-*ketamine OTF (3, 4).

In the current study, we present the results of a pharmacodynamic analysis of the effect of one and two OTFs, containing, respectively, 50 and 100 mg *S-*ketamine, administered sublingually or buccally. In the accompanying report, we showed that the *S-*ketamine OTF undergoes a large first-pass effect causing relatively high concentrations of *S-*norketamine and *S-*hydroxynorketamine (2). We studied the OTF on two end-points, nociception, by testing three distinct pain assays (pressure pain, electrical pain, and thermal pain), and drug high, one of the psychotomimetic effects of *S-*ketamine. Our main interest is the description of the pharmacodynamic effects of *S-*ketamine in the *S-*ketamine OTF. Additionally, we quantified the contribution of the *S-*ketamine metabolites in the production of antinociception and drug high. Earlier studies demonstrated that *S-*norketamine has a little analgesic effect in humans and is possibly even pro-algesic (5), while animal data indicate that *S-*hydroxynorketamine has potent analgesic and antidepressant properties (6, 7).

We performed a population pharmacodynamic analysis of the *S-*ketamine OTF in a group of healthy volunteers and built a pharmacodynamic model that incorporates the contribution of *S-*norketamine and *S-*hydroxynorketamine. Pharmacokinetic–pharmacodynamic modeling is an important tool in the development of new therapies (including new administration modes of existing therapies) to quantify the therapeutic index or utility in terms of wanted and unwanted effects and determine the contribution of metabolites.

## Methods

This report is accompanied by a report on the population pharmacokinetics of the *S-*ketamine OTF (2). Here, we describe the pharmacodynamic endpoints that were collected simultaneously with the pharmacokinetic data.

### Ethics and subjects

The protocol was approved by the Central Committee on Research Involving Human Subjects [Competent authority: Centrale Commissie Mensgebonden Onderzoek (CCMO), The Hague, the Netherlands; registration number NL75727.058.20] and the Medical Research Ethics Committee of Leiden University Medical Center (Medisch Ethische Toetsingscommissie Leiden-Den Haag-Delft, The Netherlands; identifier P20.111). It was registered at the trial register of the Dutch Cochrane Center (www.trialregister.nl) under identifier NL9267.

Healthy male and female volunteers (aged 18–45 years, body mass index ≥ 19 and ≤ 30 kg.m^−2^) were recruited. All recruited subjects gave written and oral informed consent, after which they were screened. Inclusion and exclusion criteria are given in (2). Eating, drinking, brushing teeth, or gum chewing was not allowed in the morning of the OTF application to avoid changes/variabilities in saliva pH, which could potentially affect the mucosal permeability and *S-*ketamine plasma concentration variability.

### Study design

#### S-ketamine oral thin film placement

This phase 1 study had an open-label randomized crossover design. The subjects were randomized to receive one OTF on one occasion (50 mg *S-*ketamine) and two on another (100 mg *S-*ketamine), with at least 7 days between visits. The thin film is a rectangular 4.5 cm^2^ orodispersible film containing 57.7 mg *S*-ketamine hydrochloride (*S*-ketamine HCL). The *S-*ketamine HCL is dispersed within a matrix to produce a film corresponding to 50 mg of *S-*ketamine-free base. The film(s) was/were placed either under the tongue or buccally on the mucosa. After placement of the films, the subject was not allowed to swallow for 10 min. The randomization sequence was determined by the randomization option in the Electronic Data Capture system CASTOR (www.castoredc.com). The OTFs were obtained from LTS Lohmann Therapie-Systeme AG (Andernach, Germany) and were dispensed by the pharmacy on the morning of dosing. Measurement of pharmacodynamic endpoints lasted for 6 h. For blood sampling and measurement of *S*-ketamine, *S*-norketamine, and *S*-hydroxynorketamine, see the accompanying report (2).

In all subjects, on both occasions, an intravenous *S-*ketamine infusion followed the 6-h OTF test phase and was included in the pharmacokinetic analysis to determine the *S*-ketamine bioavailability. Here, we only present the pharmacodynamic data obtained during the OTF test phase.

#### Noxious assays

Three independent pain assays, namely, thermal noxious pain, electrical noxious pain, and pressure pain, were randomly applied around predefined time intervals: *t* = 0, 10, 20, 30, 40, 60, 80, 100, 120, 150, 180, 240, 300, and 360 min after placement of the OTF(s) with 3–5 min in between tests.

Electrical pain was induced by an in-house manufactured transcutaneous electrical current stimulator (8). A constant current electrical stimulus train (stimulation at 20 Hz, pulse duration 0.2 ms) was applied to the skin over the tibial bone on the non-dominant side of the body through two surface electrodes. The location of the electrodes was such that muscle contractions did not occur. The current that induced a numerical pain rating score (NRS) of 8 on a pain scale from 0 (no pain) to 10 (worst pain imaginable) at baseline was used in the remainder of the study. The search for the correct current was performed three times before any drug administration at 5–10 min intervals in steps of 0.5 mA.

Thermal noxious stimulation was applied on the volar side of the non-dominant forearm using a 3 cm^2^ Peltier element or thermal probe of the Pathway device (Medoc Ltd., Israel) that allows computer-controlled changes in contact heat changes in steps of ± 0.5°C (8). In the current study, a heat level was chosen that at baseline caused an NRS of 8 on the above-mentioned 11-point pain scale. The correct heat level was derived from three tests at 5–10 min intervals.

Pressure pain was induced using an Algometer (FDN 200 series, Wagner Instruments Inc., Greenwich, CT) (9). Pressure pain was delivered on a 1 cm^2^ skin area between the thumb and index finger of the non-dominant hand. The device has a force capacity (± accuracy) of 200 ± 2 N (= 20 ± 0.2 kgf) and graduation of 1 N (100 gf), respectively. A gradually increasing pressure was applied and the subjects indicated when the pressure became painful (pressure pain threshold). Three tests were applied at baseline; the obtained pressure values were averaged and served as the baseline value. A researcher well-trained in this assay performed the pressure pain tests throughout the study visit days.

#### Questionnaire

The Bowdle questionnaire was taken at regular intervals to determine the effect of treatment on mental and psychotomimetic side effects (10). The timing of the questionnaires was at baseline (prior to any drug administration) and at 30-min intervals until 6 h after thin film application. In case the querying coincided with pain testing, the questionnaires were taken before pain testing. The Bowdle questionnaire allows the derivation of three factors of psychedelic ketamine effects: drug high and changes in internal and external perception. All three were measured on a visual analog score from 0 (no effect) to 10 cm (maximum effect). In the pharmacokinetic–pharmacodynamic data analysis, we included the effect of the OTF on drug high derived from the Bowdle questionnaire. The description of the effect of the *S-*ketamine OTF on the other endpoints, internal and external perception, is given in the Supplementary material.

### Population pharmacodynamic analysis

Data were analyzed in a stepwise fashion. First, the pharmacokinetic data were analyzed using a population-based approach [see (2)]. Next, the pharmacodynamic data were analyzed with individual concentration profiles of *S*-ketamine and its metabolites (based on the empirical Bayesian estimates of the pharmacokinetic parameters) as input of the sigmoid E_MAX_ pharmacodynamic models. The metabolites were assumed to be agonists or antagonists, with the total effect, EFF, modeled as:


$$\begin{array}{l} EFF=EEF\left(K\right)+EFF\left(NK\right)+EFF\left(HNK\right) \end{array}$$

with


$$\begin{array}{l} \begin{array}{l} \;EFF(K)=C_{E,K}/C_{50,K}\\ \;EFF(NK)=C_{E,NK}/C_{50,NK}\\ EFF(HNK)=C_{E,HNK}/C_{50,HNK} \end{array} \end{array}$$

or


$$\begin{array}{l} EFF={EFF\left(K\right)=\;C}_{E,K}/C_{50} \end{array}$$

with


$$\begin{array}{l} C_{50}=C_{50,K}\;\times \;\left[1+EFF\left(NK\right)+EFF\left(HNK\right)\right] \end{array}$$

and


$$\begin{array}{l} \begin{array}{l} \;EFF(NK)=\;C_{E,NK}/C_{100,NK}\\ EFF(HNK)=\;C_{E,HNK}/C_{100,HNK} \end{array} \end{array}$$

under the agonistic and antagonistic assumptions, respectively. C_E,K_, C_E,NK_, and C_E,HNK_ are the effect-site concentrations of *S*-ketamine, *S*-norketamine, and *S*-hydroxynorketamine, respectively; C_50,K_, C_50,NK_, and C_50,HNK_ are the steady-state or effect-site concentrations causing 50% of the pharmacodynamic effect; and C_100,NK_ and C_100,HNK_ are the *S*-norketamine and *S*-hydroxynorketamine concentrations causing a 100% increase of *S*-ketamine C_50_.

An effect compartment was postulated to account for the hysteresis between the *S*-ketamine plasma concentrations (and possibly its metabolites) and its effect. This effect compartment equilibrates with the plasma compartment with plasma-effect-site equilibration half-life (t½k_e0_).

The results of the electrical and thermal noxious assays were analyzed using the following inhibitory sigmoid E_MAX_ model:


$$\begin{array}{l} NRS\left(t\right)=NRS_{0}\;\times \;{\left[1+{\left(C_{E}\left(t\right)/C_{50}\right)}^{\gamma }\right]}^{-1} \end{array}$$

where NRS(t) is the NRS in response to the noxious stimulation at time t, NRS_0_ is the NRS at baseline (pre-drug condition), and γ is a dimensionless shape parameter.

For pressure pain, we assume that *S*-ketamine (and possibly its metabolites) attenuates the response to the applied noxious pressure stimulus by the inhibition of signal propagation and central nociceptive processing. As a consequence, stronger stimuli are needed before the subjects indicate that he or she experiences pain. The attenuation (A) is described by an inhibitory sigmoid E_MAX_ model (11):


$$\begin{array}{l} A={\left[1+\;C_{E}(t)/C_{50}\right)}^{\gamma }{]}^{-1} \end{array}$$

Since a response of the subjects occurs just above the response threshold, we use the following equation for the pressure pain threshold at time t:


$$\begin{array}{l} P\left(t\right)=\;P_{0}\;\times \;1/A=\;P_{0}\times \;{\left[1+\;C_{E}(t)/C_{50}\right)}^{\gamma }] \end{array}$$

where P_0_ is the baseline or pre-drug pressure that elicited a pain threshold response.

Drug high was modeled using a sigmoid E_MAX_ model:


$$\begin{array}{l} VAS\;drug\;high\;\left(t\right)=\left[E_{\max}\;\times \;C_{E}{\left(t\right)}^{\gamma }\right]/\;\left[C_{50}^{\gamma }+C_{E}{\left(t\right)}^{\gamma }\right] \end{array}$$

where C_50_ is the S-ketamine concentration that causes a drug high of 50% of E_MAX_, and E_MAX_ is the maximum possible effect on drug high (10 cm).

Data analysis was performed using NONMEM version 7.5.0 (ICON Development Solutions, Hanover, MD, USA). Inter-occasion variability (ν^2^) was determined for baseline values only, as we assumed that other parameter values would not differ between the two occasions and were drug-dose independent. Determining whether the metabolites contributed to the measured effect and the level of significance of model parameters were based on the log-likelihood criterion (-2LL; a decrease of more than 6.6 is significant at the *p* < 0.01 level for one additional parameter). The goodness of fit was based on the visual inspection of the model fits and goodness of fit plots (individual predicted vs. measured, individual weighted residuals vs. time, and normalized prediction discrepancy error vs. time). Additionally, visual predictive checks (PVCs) were generated to ensure that the models were able to reproduce the data used for model building. Although no pharmacokinetic differences were observed in the sublingual and mucosal applications, we compared the location of the application on the pharmacodynamic parameter estimates.

## Results

Twenty subjects participated in the study to receive, in random order, a single OTF (50 mg) or, on a second occasion, two OTFs applied simultaneously (100 mg) sublingually (*n* = 15) or on the buccal mucosa (*n* = 5). One subject participated only once (receiving 100 mg *S-*ketamine) due to side effects experienced from the intravenous *S-*ketamine infusion. Since no differences were observed in the sublingual and mucosal application regarding pharmacokinetics and pharmacodynamics of S-ketamine and its metabolites, we combined the two in the pharmacokinetic and pharmacodynamic model analyses. The mean age of the volunteers was 24 years (with range 19–32 years), mean weight 73 kg (53–93 kg), and mean body mass index 23 kg/m^2^ (19–27 kg/m^2^) with an equal number of men and women participating (10/10).

**Figure 1:**
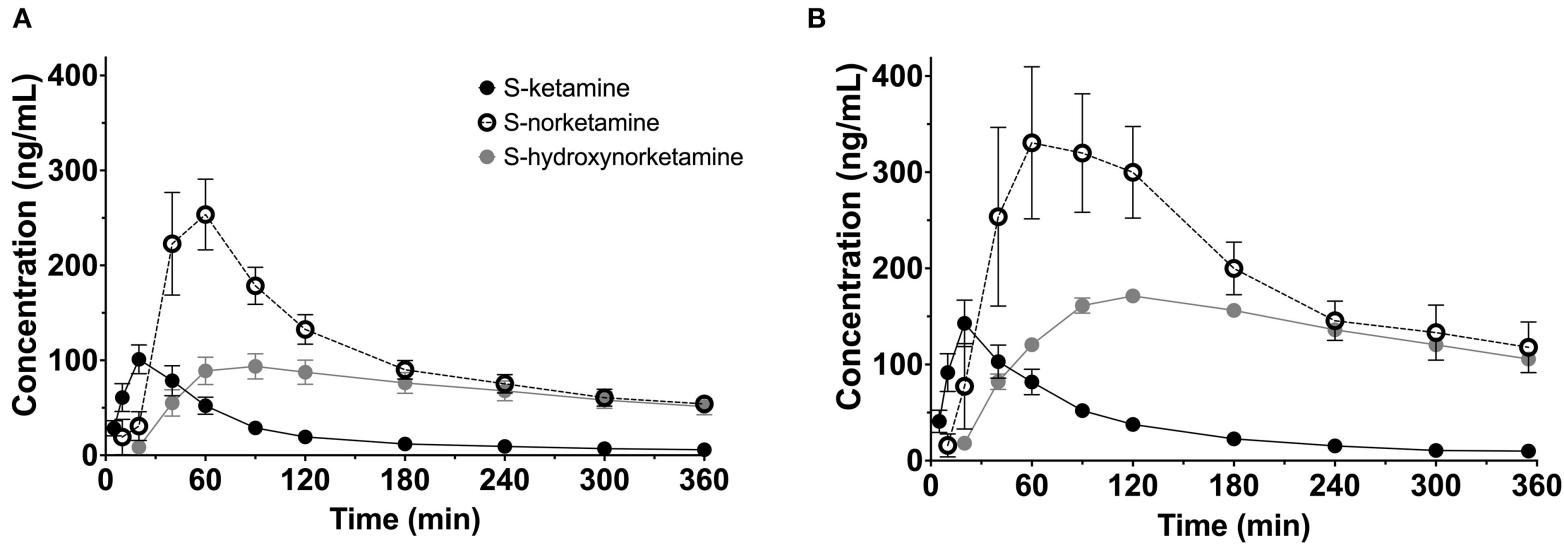
Pharmacokinetic data. Average plasma concentrations ± 95% confidence intervals for *S*-ketamine, *S*-norketamine, and *S*-hydroxynorketamine following administration of 50 mg **(A)** and 100 mg **(B)** *S-*ketamine oral thin film.

**Figure 2:**
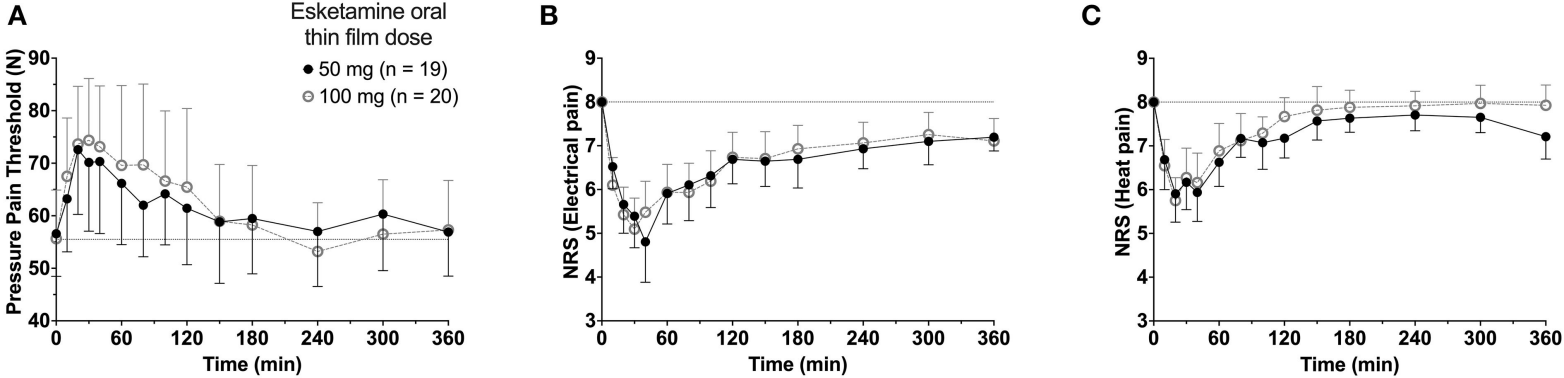
Antinociceptive data. The effects of the 50 mg and 100 mg *S-*ketamine oral thin film on pain responses for the three pain assays, pressure pain threshold **(A)**, electrical pain numerical rating scale **(B)**, and heat pain numerical rating scale **(C)**. Date are mean ± 95% confidence interval.

To summarize the pharmacokinetic data that stands at the basis of the pharmacodynamic analyses, we give the average plasma concentrations for *S*-ketamine, *S*-norketamine, and *S*-hydroxynorketamine following 50 and 100 mg *S-*ketamine OTF in Figure 1. It shows the large first-pass effect, with relatively high concentrations of the S-ketamine metabolites. No serious adverse events occurred during the study [see (2) for a description of adverse events]. The effects of the 50 and 100 mg *S-*ketamine OTF on pain responses are given in Figure 2 for the three assays: electrical pain, heat pain, and pain pressure. The data indicate that the OTF produces antinociception in all three assays, but irrespective of the pain assay a clear dose-response relationship was absent. Among subjects, pain relief was most variable in the pain pressure test compared to the other pain test with pain relief lasting 2 h (heat pain test) or longer (electrical pain test). Comparing Figures 1, 2 gives rise to the suggestion that the *S-*ketamine effect is pharmacokinetically driven, i.e., the pain responses closely follow the *S-*ketamine concentration profile, without any effect of the metabolites. In **Figures 4A,B**, the individual median drug high visual analog scores are given in gray and red, respectively. The peak median effect is higher for the 100 mg *S-*ketamine OTF compared to the lower dose OTF.

### Population pharmacodynamic analysis

**Figure 3:**
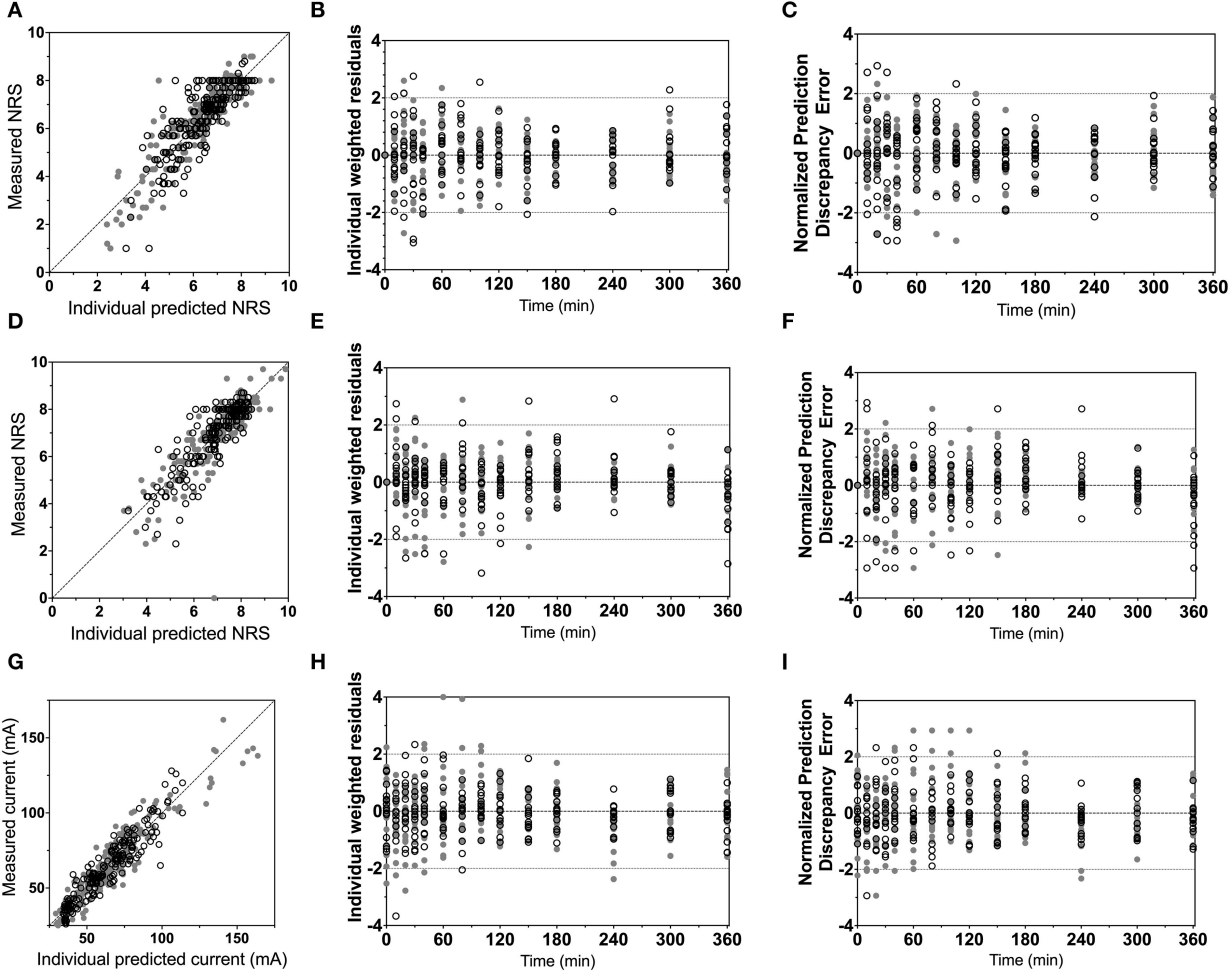
Goodness-of-fit plots. **(A–C)** Electrical pain numerical rating scale; **(D–F)** heat pain numerical rating scale; **(G–I)** pressure pain threshold. **(A,D,G)** Measured vs. individual predicted; **(B,E,H)** individual weighted residuals vs. time. **(C,F,I)** Normalized prediction discrepancy errors vs. time. Dashed lines are the 95% confidence intervals. Open circles denote data from the 50 mg *S-*ketamine oral thin film and closed circles data from the 100 mg *S-*ketamine oral thin film.

**Figure 4:**
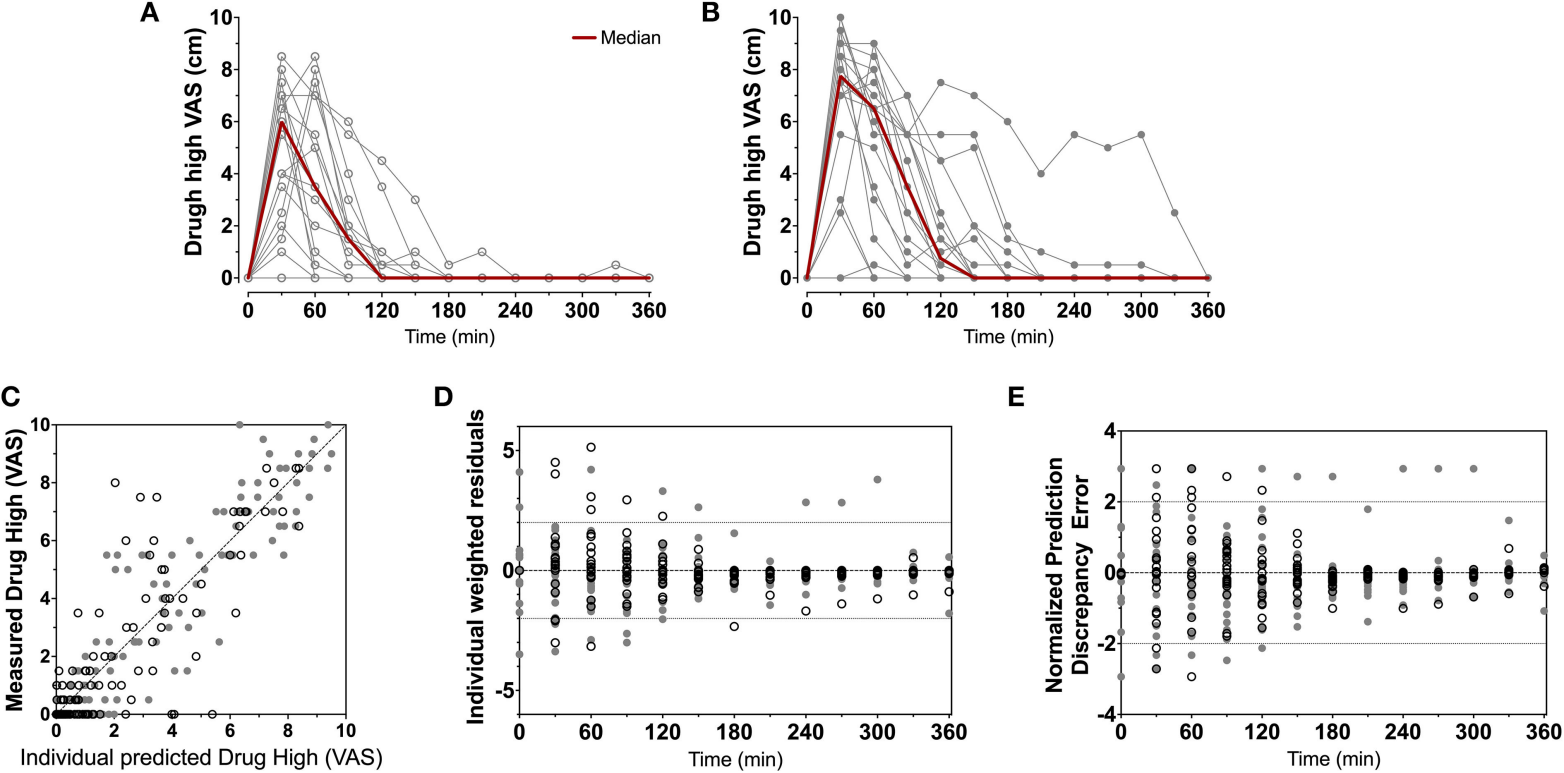
Drug high data. Individual and median drug high visual analog scores following the 50 mg **(A)** and 100 mg **(B)** *S-*ketamine oral thin film. **(C–E)** Goodness-of-fit plots, **(C)** measured vs. individual; **(D)** individual weighted residuals vs. time; **(E)** normalized prediction discrepancy errors vs. time. Dashed lines are the 95% confidence intervals. Open circles denote data from the 50 mg *S-*ketamine oral thin film and closed circles data from the 100 mg *S-*ketamine oral thin film.

**Figure 5:**
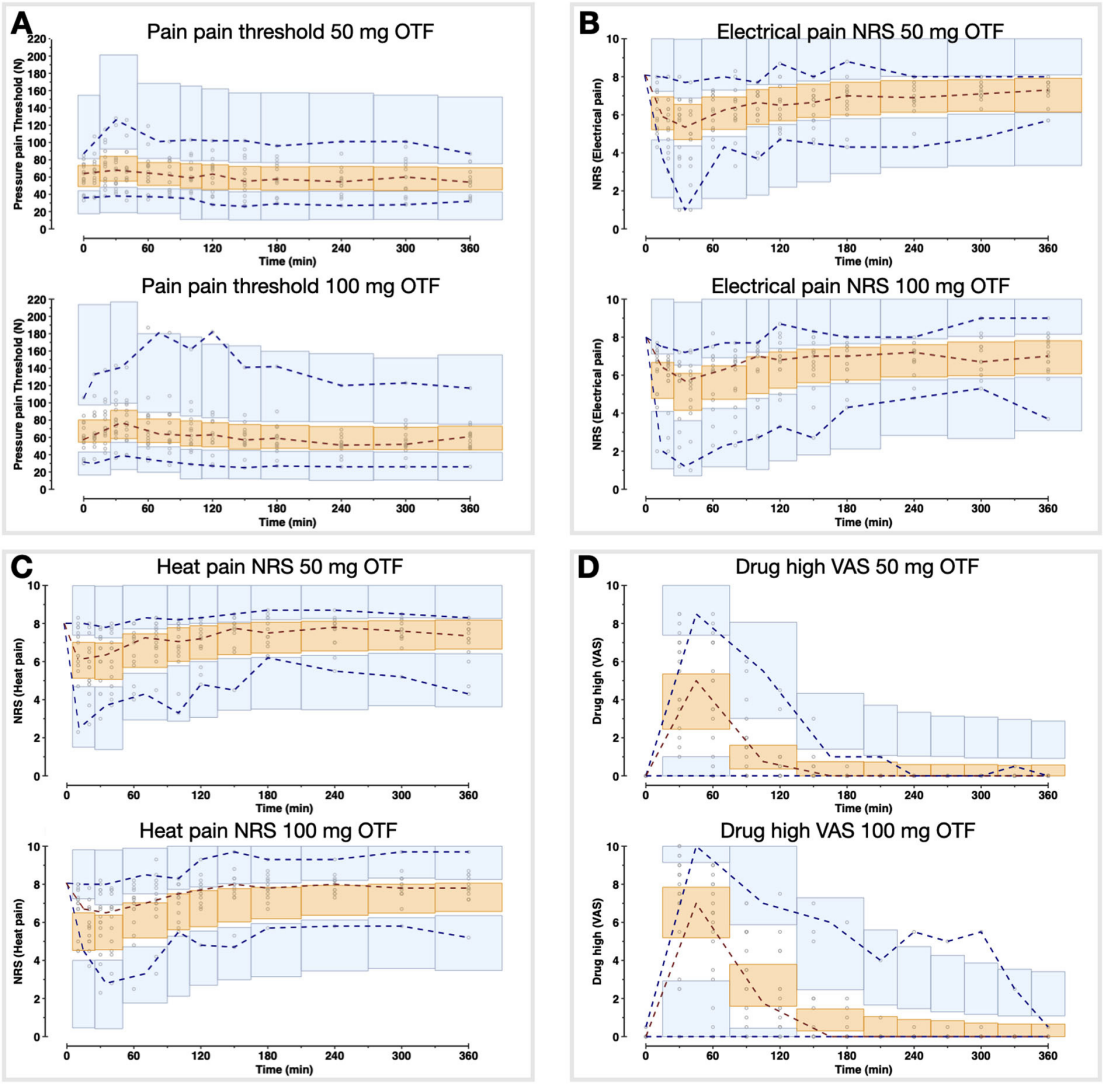
Visual predictive checks of the pharmacodynamic model for the two *S-*ketamine OTF doses. The circles are the measured data points; the broken lines are the observed percentiles (dark red: median, dark blue: 2.5th and 97.5th percentiles); the bins are the 95% confidence intervals of simulated percentiles (orange bins: median, blue bins: 2.5th and 97.5th percentiles). **(A)** Pain threshold; **(B)** Electrical pain numerical rating scale (NRS); **(C)** Heat pain NRS; **(D)** Drug high visual analog scale (VAS).

**Figure 6:**
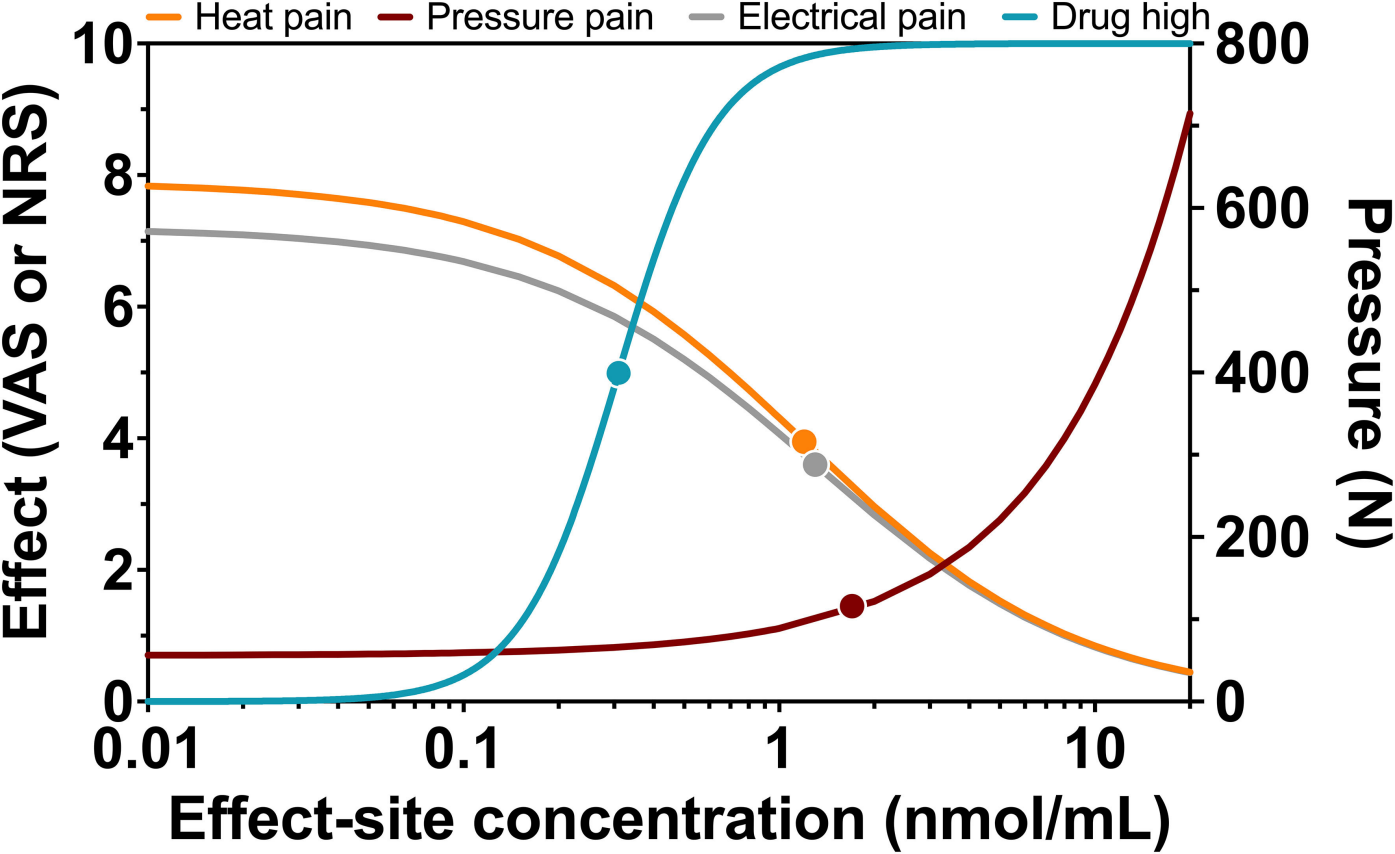
Steady-state concentration-effect relationships for heat pain, pressure pain, electrical pain, and drug high. Relationship between steady-state or effect-site *S-*ketamine plasma concentrations and effect with dots representing C_50_ values or half-effect concentration values.

Population parameters estimates are given in Table 1, the best median and worst fits (based on *R*^2^) for all 4 endpoints are given in Supplementary Figure 1. The goodness of fit plots are given in Figures 3, 4, panels C-E. PVCs, comparing observations with model predictions, are given in Figure 5. Inspection of the fits, goodness of fit plots, and PVCs indicate that the PKPD models adequately describe the data with no differences between the 50 and 100 mg OTF-related data (in Figures 3, 4; open circles 50 mg *S-*ketamine OTF, closed circles 100 mg *S-*ketamine OTF). No contribution of either *S*-norketamine or *S*-hydroxynorketamine could be detected, i.e., EFF(NK) and EFF(HNK) approached zero. Consequently, the antinociceptive and drug high effects are attributed solely to S-ketamine. S-ketamine potency was about 3- to 5-fold lower for antinociception than for drug high: C_50_ 1.2–1.7 nmol/mL vs. 0.3 nmol/mL for the nociceptive tests and drug high, respectively. The onset/offset of the *S*-ketamine effect was similarly fast for all tests, nociceptive and drug high, and ranged from a value not different from zero (Table 1), indicative of an instantaneous effect to 5 min. In Figure 6, the steady-state or effect-site concentration-effect relationships are given for the four pharmacodynamic endpoints. The dots in the figure depict the C_50_ values. No effect of the location of the OTF on parameter estimates was observed (*p* > 0.05).

**Table 1:** Pharmacodynamic parameter estimates.

	**Estimate ±SEE**	**ω^2^ ±SEE**	**ν^2^ ±SEE**
**Electrical pain NRS (0–10)**			
Baseline NRS	7.2 ± 0.2		0.025 ± 0.010
t½k_e0_ (min)	3.4 ± 0.4	1.13 ± 0.81	
C_50_ (nmol/mL)	1.3 ± 0.2	1.01 ± 0.32	
γ	1 (FIX)		
σ	0.75 ± 0.07		
**Heat pain NRS (0–10)**			
Baseline NRS	7.9 ± 0.2		0.011 ± 0.006
t½k_e0_ (min)	-		
C_50_ (nmol/mL)	1.2 ± 0.32	0.57 ± 0.27	
γ	1 (FIX)	0.57 ± 0.31	
σ	0.66 ± 0.08		
**Pressure pain**			
Baseline (N)	56 ± 5	0.09 ± 0.03	0.03 ± 0.01
t½k_e0_ (min)	-		
C_50_ (nmol/mL)	1.7 ± 0.29	0.27 ± 0.11	
γ	1 (FIX)		
σ (N)	9.26 ± 1.18		
**Drug high VAS (0–10 cm)**			
t½k_e0_ (min)	5.4 ± 0.2	1.1 ± 0.69	
C_50_ (nmol/mL)	0.31 ± 0.03	0.16 ± 0.08	
γ	2.8 ± 0.3	0.15 ± 0.05	
σ	0.89 ± 0.10		

NRS, numerical rating scale; t½k_e0_ is blood-effect-site equilibration half-life; C_50_ is the concentration at steady-state causing 50% effect; γ is a shape parameter and σ a measure of residual noise; SEE is the standard error of the estimate.

## Discussion

The main findings from our population pharmacodynamic modeling study of an *S-*ketamine OTF are summarized as follows: (1) the sublingual and buccal 50 and 100 mg *S-*ketamine OTFs are antinociceptive with effects lasting at least 2 h; (2) the onset of effect is rapid with peak effects within 30–45 min; (3) drug high had a peak effect at 30 min and lasted at least 2 h; (4) *S*-ketamine potency was lower for antinociception than for drug high by a factor of 3–5; (5) there were no contributions of *S*-norketamine and *S*-hydroxynorketamine to the antinociceptive or drug high *S-*ketamine effects detected in our model.

A clear dose-response relationship was not observed in the nociceptive data. For all three pain assays, the effect of doubling the dose of the OTF did not produce a significant increase in antinociception. Several mechanisms may be involved: (i) this may be related to a 20% lower S-ketamine bioavailability for 100 mg OTF compared to 50 mg OTF (bioavailability 50 mg = 29% vs. 100 mg 23%) (2); (ii) as observed in Figure 6, for electrical and heat pain, the effect-site concentration-effect curve becomes ultimately flat at high concentrations; for pressure pain, the slope of the response is initially, not particularly, steep, and some overlap in the response data may be expected; (iii) the high concentrations of the measured and non-measured metabolites may have an antagonistic effect on the antinociceptive response (5). High concentrations of *S*-norketamine and *S*-hydroxynorketamine were observed after 50 and 100 mg *S-*ketamine OTFs due to the high first-pass effect. We earlier showed that *S*-norketamine counteracts the effects of *S*-ketamine (5), but see below; (iv) possibly some noise in the data and variability in the day-to-day analgesic drug efficacy may have caused the overlap of antinociceptive response; (v) since we applied several noxious stimuli in a relatively short period of time to the volunteers, this may have altered the discriminatory ability of the nociception signaling pathways. Such an effect was earlier observed for volunteers treated with an opioid (12); (vi) and finally, theoretically, there may be a ceiling in the ability of the nociceptive assays at higher drug doses. In a *post-hoc* analysis, we observed that the separately estimated C_50_s following the two *S-*ketamine OTFs did not differ, indicative that the absence of a dose-dependency of antinociceptive effect is probably related to the following items: (i) reduced bioavailability for the higher dose OTF, (ii) flat concentration-effect relationship, and (iii) lesser discriminatory ability of the pain signaling pathways when multiple stimuli are administered. An effect of metabolites on the antinociceptive and drug high responses is further discussed below.

### Norketamine and hydroxynorketamine

Due to the large first-pass effect, plasma concentrations of *S*-norketamine and *S*-hydroxynorketamine were relatively high compared to intravenous ketamine administration [(2) and Figure 1]. Our modeling approach detected no contribution of these two *S*-ketamine metabolites to the antinociceptive and drug high effects of the *S-*ketamine OTF. Animal studies do show an antinociceptive effect from both ketamine and norketamine (13, 14), while we earlier observed a small negative contribution of *S*-norketamine to the antinociceptive and hemodynamic effects of *S*-ketamine in healthy volunteers (5). We argued that this is one of the main reasons for the observation of pain facilitation after ketamine treatment when norketamine concentrations exceed ketamine concentrations in plasma. Such observations are sometimes observed both clinically and in experimental studies (15–17). The absence of a negative *S*-norketamine contribution to the antinociception from the OTF suggests that *S*-norketamine has either no antinociceptive effect in humans or the effect is small and was not detected from the noise in the data in our current study. Still, the absence of treatment arms that received *S*-norketamine precludes a definite conclusion regarding the effect of either metabolite on the pharmacodynamic responses in our study. For now, we cautiously infer from our modeling approach that *S*-norketamine has no or just little effect on either analgesia or drug high in human volunteers. The excitatory phenomena observed after ketamine infusion in other studies may then be related to the rebound activation of *N*-methyl-D-aspartate receptor and non-*N*-methyl-D-aspartate receptor glutamate receptors from accumulated excitatory amino acids in the synaptic cleft (17).

In an animal study, Kroin et al. (7) showed earlier that (*2R,6R*)-hydroxynorketamine is an efficacious analgesia in mice. In three pain models, nerve-injury neuropathic pain, tibia fracture complex regional pain syndrome, and plantar incision postoperative pain, (2R,6R)-hydroxynorketamine was effective and superior to ketamine in terms of efficacy and side effect profile. Hence, we anticipated a long-term analgesic effect from *S*-hydroxynorketamine in our model with sustained and relatively high *S*-hydroxynorketamine concentrations in plasma. While after intravenous *S*-ketamine, the ratio of peak concentration *S*-hydroxynorketamine to *S*-ketamine equals 0.3 (3), and this ratio ≈ 1 after the application of the *S-*ketamine OTF, irrespective of dose (Figure 1). The absence of an *S*-hydroxynorketamine contribution may be dose-related (i.e., at a higher concentration an effect may become visible), related to the stereoselectivity in the effect of hydroxynorketamine [*S*-hydroxynorketamine in our study vs. *R*-hydroxynorketamine in the study of Kroin et al. (7)], and finally it may be due to the cancellation of an antinociceptive effect from *S*-hydroxynorketamine by a pronociceptive rebound effect from the accumulated excitatory amino acids following the decline in *S*-ketamine plasma concentration and reduced blockade of NMDA glutamatergic receptors (16). This later mechanism would then suggest that *S*-ketamine and *S*-hydroxynorketamine act at different receptor systems to induce analgesia. Zanos et al. showed indeed that (*2R*,*6R*)-hydroxynorketamine, but not ketamine, acts at the non-NMDA glutamate α-amino-3-hydroxy-5-methyl-4-isoxazolepropionic acid (AMPA) receptor (9). Moreover, a recent study from Bonaventura et al. (18) showed that (*2R,6R*)-hydroxynorketamine displays minimal brain uptake and rapid clearance from the brain, without any affinity for opioid receptors or any other known ketamine targets. Evidently, we need to consider the stereoselective effects of ketamine's metabolites. We argue therefore that, similar to *S-*norketamine, *S-*hydroxynorketamine needs to be administered to humans, in future studies, to quantify its analgesic effect.

### Drug high vs. analgesia

For drug high, a dose dependent *S*-ketamine effect was observed (Figures 4A,B), without any contribution from its metabolites. Relative to the *S*-ketamine antinociception, *S*-ketamine was about 3–5 times more potent in producing its drug high effects with a C_50_ (*S*-ketamine concentration causing a drug high of 5 on an 11-point scale from 0 to 10) of 0.31 nmol/ml. Interestingly, earlier studies showed that the racemic ketamine C_50_ for drug high is at least a factor 2 greater than that of *S*-ketamine, indicative of a greater *S*-ketamine potency compared to the *R*-isomer and the racemic mixture in producing drug high effects with some studies finding that *R-*ketamine does not produce any psychotypical effects (19, 20).

Drug high effects were predominantly present during the analgesic period, suggestive of a connection between drug high and pain relief. A connection or association between the various ketamine endpoints such as analgesia and its psychotomimetic effects has been a matter of debate and has recently been refuted (20–22). However, the current data set and earlier studies from our laboratory support an intricate association between analgesia and psychotomimetic side effects (20). What this means is still unclear. It may relate to a similar site of action within the brain, or more probably, a connection between distinct brain areas that fire together upon exposure to ketamine. The latter would cause similar dynamics of the response (i.e., with similar onset/offset times), although potency between the two endpoints may differ. We plan further studies to increase our insights into this matter.

## Conclusion

In this pharmacokinetic–pharmacodynamic modeling study, we tested the antinociceptive and drug high effects of an *S-*ketamine OTF. The OTF was safe, and the side effects were related to ketamine itself and not to the thin film. Despite low bioavailability (on average 26%), the *S-*ketamine OTF produced potent antinociceptive responses in all three assays lasting 2–6 h, effects that were related to *S*-ketamine and not to its two metabolites, *S*-norketamine and *S*-hydroxynorketamine. The clinical indication of the OTF is primarily treatment of acute pain, for example in the emergency room, in the ambulance following acute trauma, or for wound dressing. Additionally, we see a place for the *S-*ketamine OTF in the treatment of severe (cancer and non-cancer) breakthrough pain. However, further clinical studies are needed to address this issue.

## Data availability statement

The data are available from the authors after agreement has been obtained regarding purpose of analysis and protocol. Requests to access the datasets should be directed to a.dahan@lumc.nl.

## Ethics statement

The studies involving human participants were reviewed and approved by METC-LDD. The patients/participants provided their written informed consent to participate in this study.

## Author contributions

AD, MV, MN, PM, and FH were involved in the design of the study. PM, ML, TD, MN, RS, and AD performed experiments and contributed to the data collected. AD and EO designed the statistical analysis and performed data analysis. MN and MV supervised the project. PS, EO, MV, ML, TD, PM, FH, RS, MN, and AD contributed to data interpretation. AD wrote the initial draft of the manuscript. All authors contributed to data interpretation, final drafting of the manuscript and approved the submitted version.

## Funding

This study was supported by LTS Lohmann Therapie-Systeme AG, Andernach, Germany and institutional funds. LTS Lohmann Therapie-Systeme AG was not involved in the study design, collection, analysis, interpretation of data, the writing of this article or the decision to submit it for publication.

## Conflict of interest

Authors PM and FH were employees of LTS Lohmann Therapie-Systeme AG, Andernach, Germany. The remaining authors declare that the research was conducted in the absence of any commercial or financial relationships that could be construed as a potential conflict of interest.

## Publisher's note

All claims expressed in this article are solely those of the authors and do not necessarily represent those of their affiliated organizations, or those of the publisher, the editors and the reviewers. Any product that may be evaluated in this article, or claim that may be made by its manufacturer, is not guaranteed or endorsed by the publisher.
