# Supplementary material for: S-Ketamine oral thin film—Part 2: Population pharmacodynamics of S-ketamine, S-norketamine and S-hydroxynorketamine
Source: Front Pain Res (Lausanne). 2022 Aug 11;3:946487. doi: 10.3389/fpain.2022.946487 (PMC9402896; doi:10.3389/fpain.2022.946487)
Supplement: Supplementary file 1 [file Data_Sheet_1.PDF]

Supplemental materials to

S-ketamine oral thin film – Part 2: population pharmacodynamics of *S*-ketamine, *S*-norketamine and *S*-hydroxynorketamine

Pieter Simons, Erik Olofsen, Monique van Velzen, Maarten van Lemmen, Tom van Dasselaar, Patrick Mohr, Florian Hammes, Rutger van der Schrier, Marieke Niesters, Albert Dahan

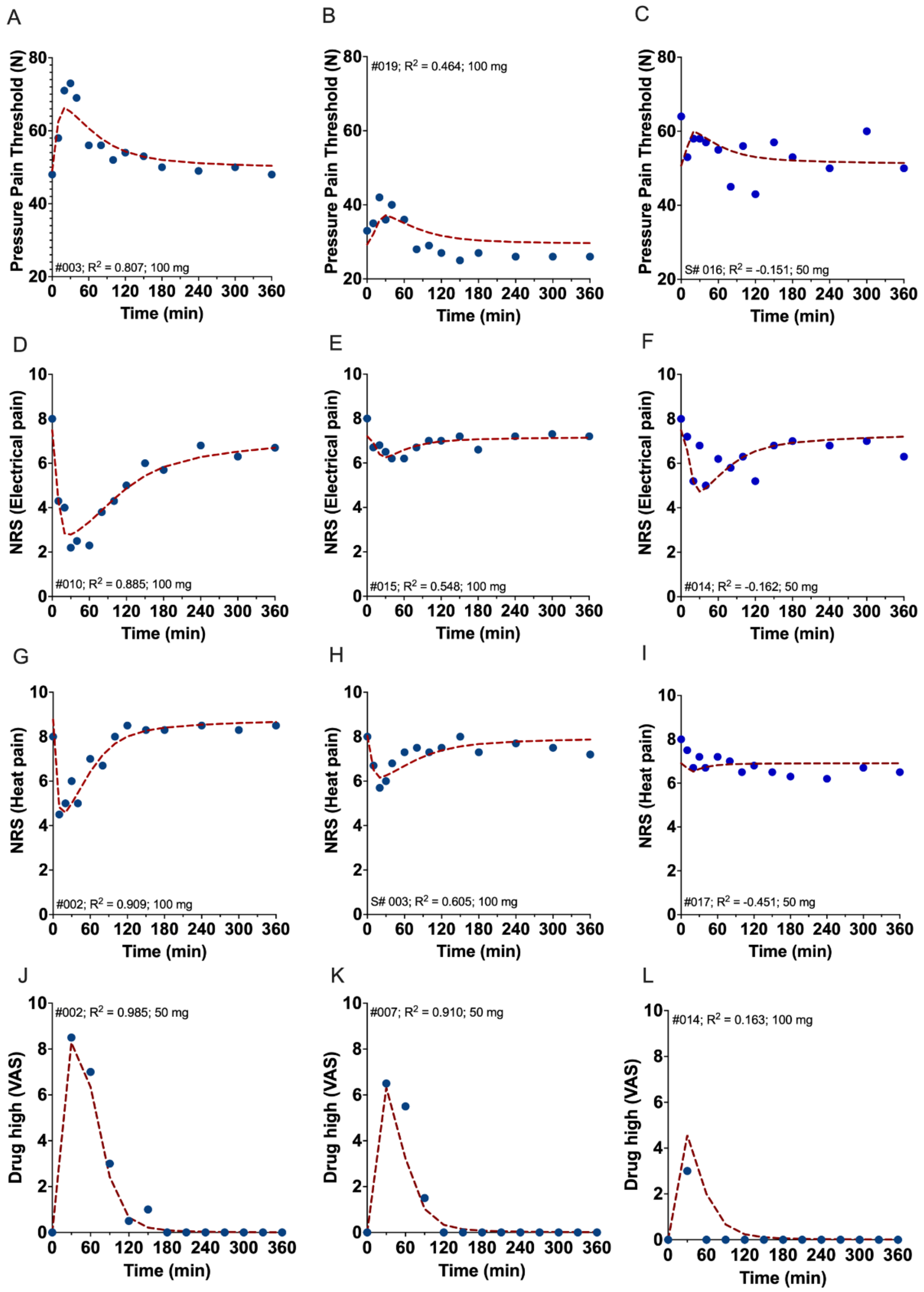

Supplemental Figure 1. Best, median and worst fits for pressure pain threshold (**A-C**), electrical pain numerical rating score (NRS) (**D-F**), heat pain NRS (**G-I**) and drug high visual analogue scale (VAS; **J-L**). Blue dots are measured data, red line are the estimated data fits. Subject number (#), R^2 and dose are given in the panels. **A, D, G** and **J** are best fits, **B, E, H** and **K** are median fits and **C, F, I** and **L** are worst fits.

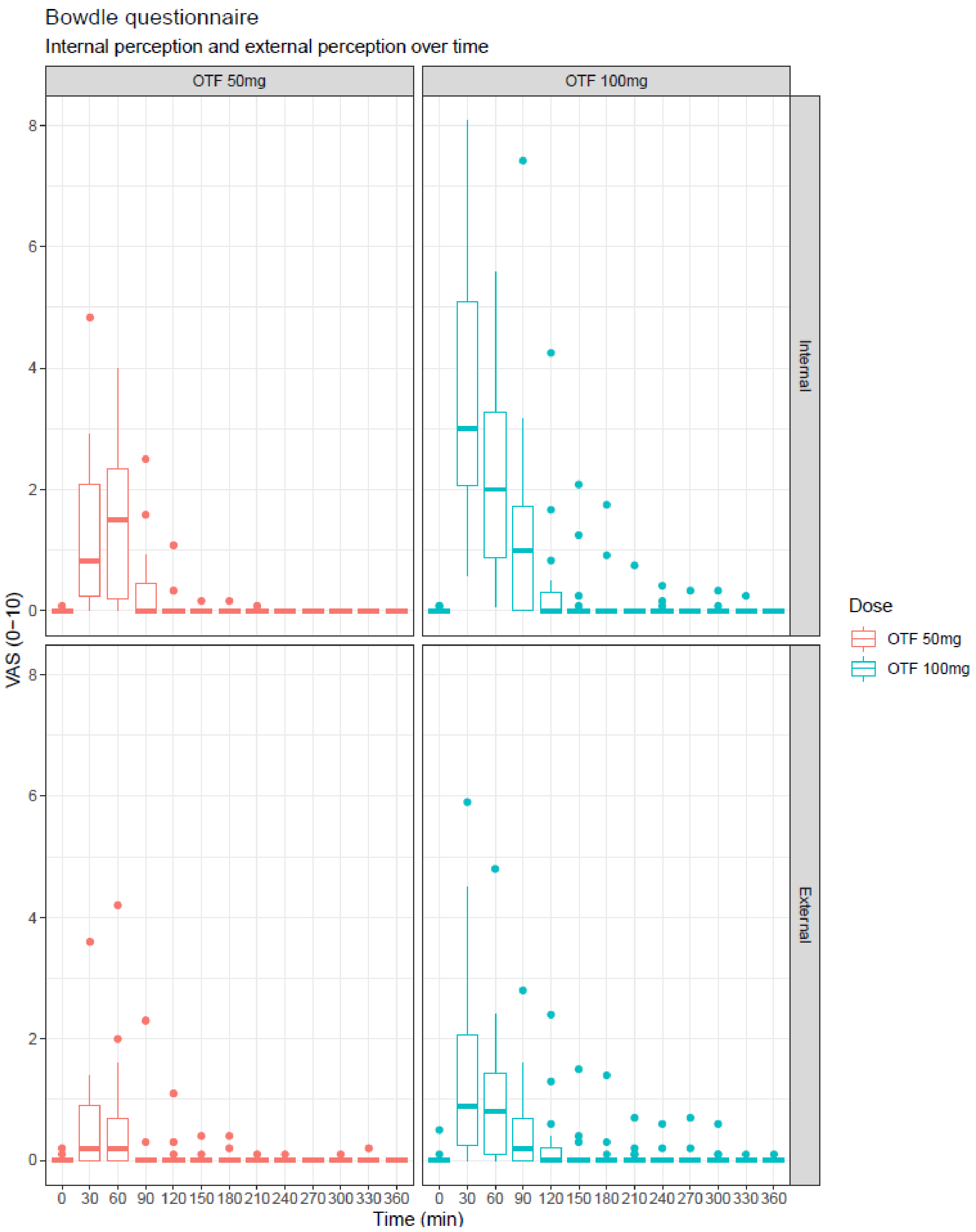

Supplemental Figure 2: Boxplots of the effect of the *S*-ketamine oral thin film on internal and external perception derived from the Bowdle questionnaire. Data are given for the application of the 50 and 100 mg oral thin film.
